# Hypogenic Pectoralis Major Muscle Associated With Complete Agenesis of the Pectoralis Minor Muscle: A Cadaveric Case Report

**DOI:** 10.7759/cureus.78589

**Published:** 2025-02-05

**Authors:** Soumya Sharma, Grace Earl, Adel Maklad, Wendy Lackey-Cornelison, Hamoun Delaviz

**Affiliations:** 1 Department of Medical Education, University of Toledo College of Medicine & Life Sciences, Toledo, USA

**Keywords:** anatomical variation, educational anatomy, hypogenic muscle development, pectoralis major, pectoralis minor

## Abstract

The pectoralis major (PM) and pectoralis minor (PMi) are muscles located in the anterior chest wall. The PM is a fan-shaped muscle composed of the clavicular and sternocostal heads. Typically, the clavicular head originates from the anterior surface of the medial half of the clavicle. The sternocostal head, located just inferior to the clavicular head, originates from the anterior surface of the sternum, superior six costal cartilages, and aponeurosis of the external oblique muscle. The PMi lies deep into the PM, positioned on top of the rib cage. Innervation of these muscles is provided by the medial and lateral pectoral nerves. Pectoralis muscle anomalies can occur due to congenital reasons, genetic factors, or developmental changes. This paper explores one such anomaly. During a routine educational cadaveric dissection at the University of Toledo College of Medicine, asymmetrical PM muscles were identified in a 98-year-old male. The dissection revealed that the sternocostal head of the left PM was not fully developed. Additionally, the left PMi muscle was missing, and the left medial pectoral nerve was absent. Anomalous development of the PM is often associated with other musculoskeletal developmental defects, and the clinical presentation can vary depending on the involvement of structures. The PMi acts as a surgical landmark and can also be used as a myo-cutaneous flap in reconstructive surgeries. The PMi tendon is often used in the rotator cuff and acromioclavicular joint repairs. Understanding the anomaly presented in this case report will help physicians manage future cases of anomalous PM and PMi in their patients.

## Introduction

The pectoralis major (PM) muscle is the largest muscle of the anterior chest wall and typically consists of two heads: the clavicular head and the sternocostal head. PM’s clavicular head typically originates from the anterior surface of the medial half of the clavicle, while the sternocostal head originates from the anterior surface of the sternum, superior six costal cartilages, and aponeurosis of the external oblique muscle [1]. The PM muscle plays a significant role in the adduction and medial rotation of the humerus. Additionally, this muscle helps pull the scapula anteriorly and inferiorly. The clavicular head helps flex the humerus, while the sternocostal head extends the humerus. When both heads of the PM muscle work together, they cause adduction and medial rotation of the humerus [2]. The lateral pectoral nerve innervates the clavicular head, while the medial pectoral nerve innervates the sternocostal head. The thoracoacromial trunk supplies PM and the venous drainage is done by the pectoral branch of the thoracoacromial vein, which drains into the subclavian vein [3].

The pectoralis minor (PMi) muscle lies deep to the PM and superficial to the ribs on the chest [3]. PMi typically originates from the coracoid process of the scapula and inferiorly attaches to ribs 3-5 [3]. PMi does not serve a major functional role. However, it can protract and depress the scapula while also helping to stabilize the shoulder. It can also aid in downward rotation and anterior tilting of the scapula [4]. The medial pectoral nerve is responsible for the innervation of the PMi and the lower half of the PM muscle. The medial pectoral nerve branches off the brachial plexus, usually the medial cord (in 49.3% of cases) or the anterior division of the lower trunk (43.8% of cases) [5]. The thoracoacromial or lateral thoracic arteries provide blood supply to the PMi. In 77% of cases, the flow is from a single artery, not a combination. Vascular drainage is variable but usually matches the arterial supply. Regardless, venous damage from the PMi travels to the axillary vein [6].

If PMi is absent, the patient may experience difficulty in stabilizing the scapula during certain shoulder movements which can manifest as shoulder instability or discomfort during physical activities [7]. Bond investigated the effects of the PMi’s absence on scapular kinematics during overhead activities [7]. The study emphasized that a shortened or missing PMi leads to increased scapular protraction and diminished upward rotation, both essential for efficient overhead movement [7].

In this study, we present a case describing an anomalous PM and an absent PMi. Clinically, a patient with this anomaly should have a reduced range of motion in the shoulder joints, particularly in movements of shoulder adduction and flexion [8]. Still, compensatory movements in the scapula might overcome this effect [8]. This compensatory position of the scapula can lead to shoulder pain or changes in posture over time [8]. Overall functional changes should thus be minimal [8].

Variations in its origin, insertion, and innervation can occur, leading to anomalous presentations. Understanding these variations is important, as they can have functional implications leading to impairments in shoulder movement, postural alignment, and/or muscle strength. Understanding these variations is important for clinicians, particularly in the settings of breast reconstruction, shoulder repairs, trauma management, or in the usage of muscle flaps to optimize favorable outcomes.

## Case presentation

**Figure 1:**
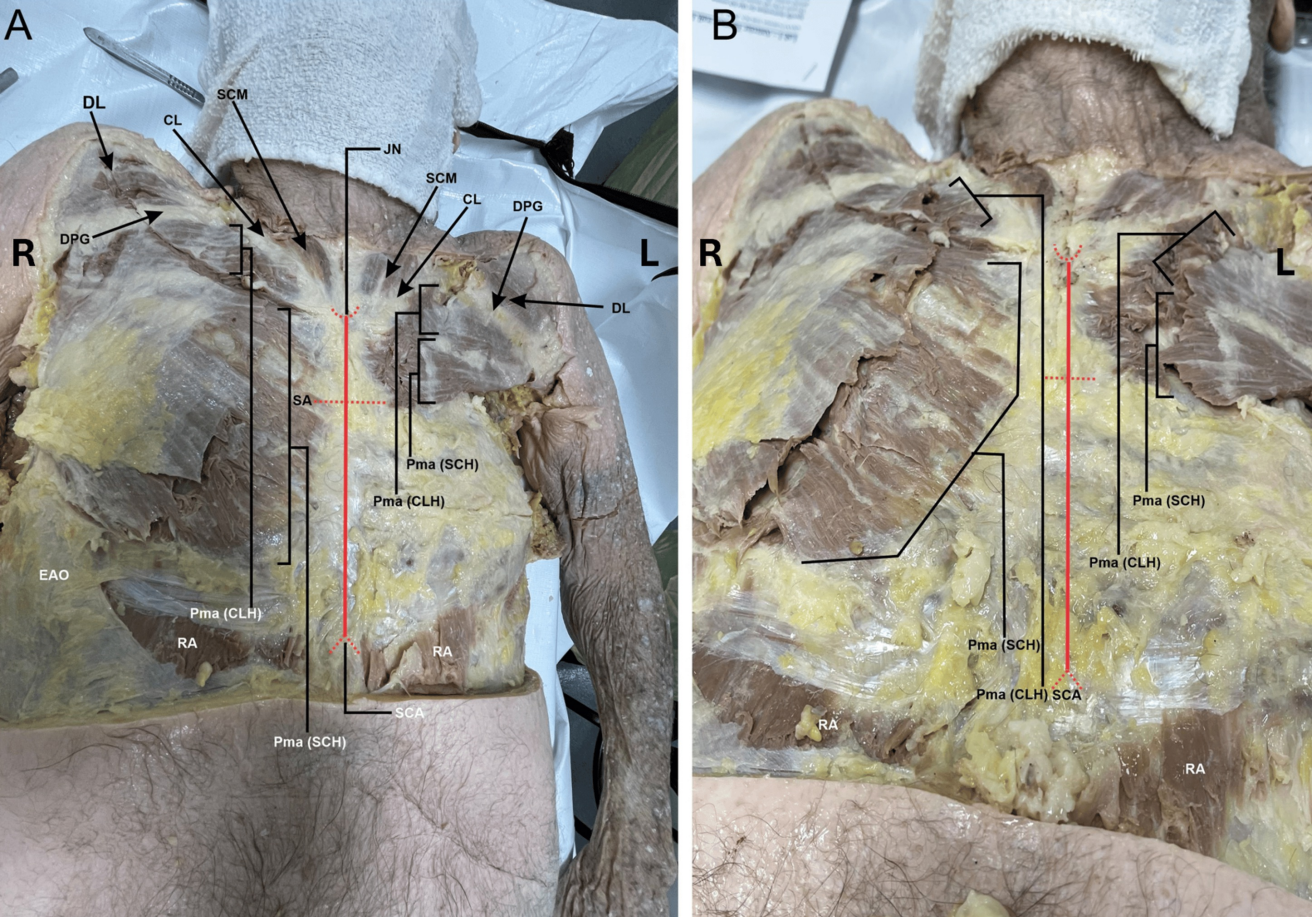
Superficial dissection of the pectoral region after removal of the skin and superficial fascia. 1A) A zoomed-out view of the anterior thorax with right and left pectoralis major muscles. 1B) Zoomed-in view of the pectoral region showing the size disparity of the right and left pectoralis major muscles. CL: clavicle; DL: deltoid (Clavicular head); DPG: deltopectoral groove; EAO: external abdominal oblique; JN: jugular notch; L: left; Pma (CLH): clavicular head of pectoralis major; Pma (SCH): sternocostal head of pectoralis major; R: right; RA: rectus abdominalis; SA: sternal angle; SCA: subcostal angle; SCM: sternocleidomastoid

**Figure 2:**
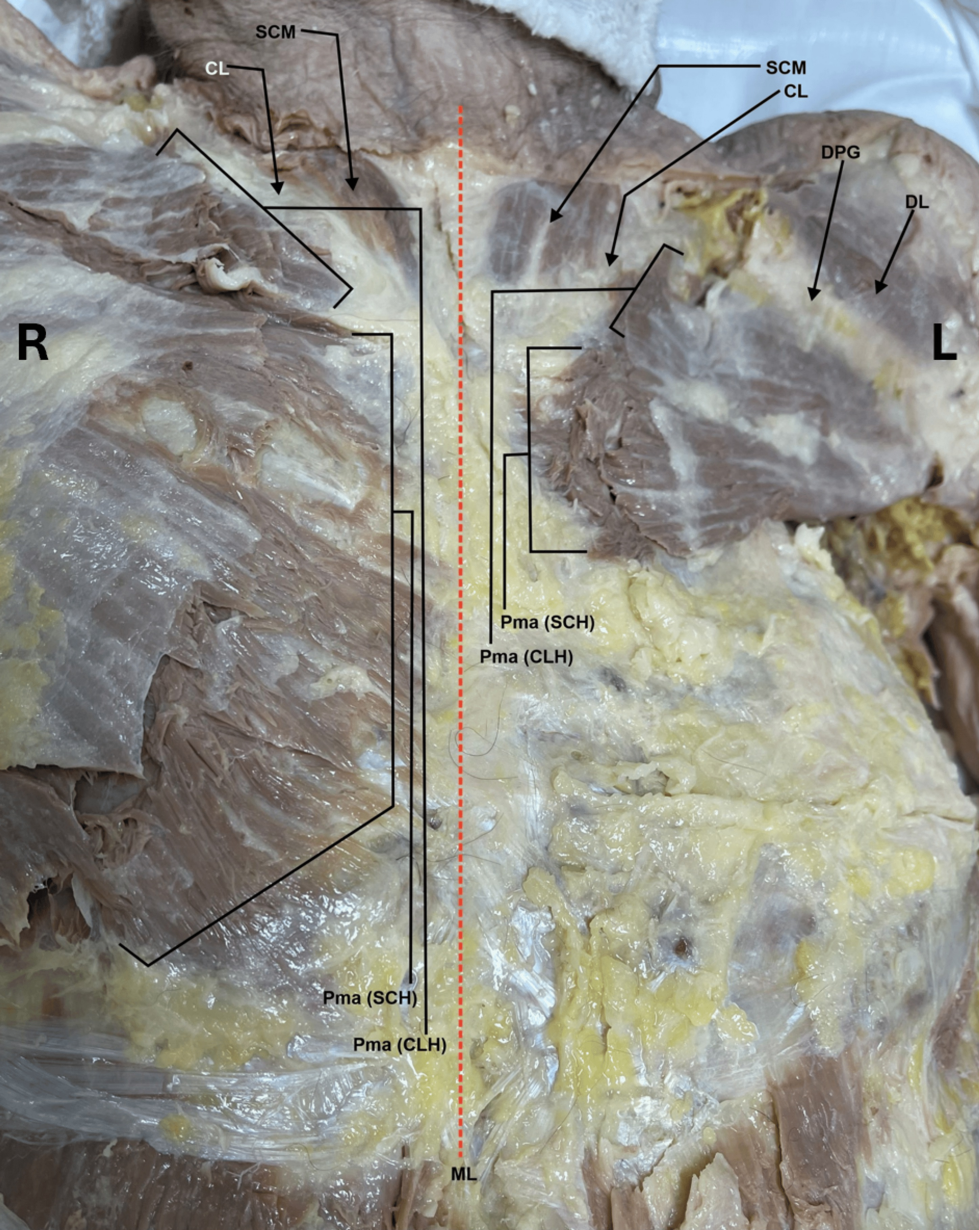
A zoomed-in view of the right and left pectoralis major near the region of the midline (dashed red line) showing more clearly the origin of the right and left pectoralis major. Note that the sternocostal head of the left pectoralis major was very rudimentary compared to the expansive sternocostal head of the right pectoralis major. CL: clavicle; DL: deltoid (Clavicular head); DPG: deltopectoral groove; L: left; ML: midline; Pma (CLH): clavicular head of pectoralis major; Pma (SCH): sternocostal head of pectoralis major; R: right; SCM: sternocleidomastoid

**Figure 3:**
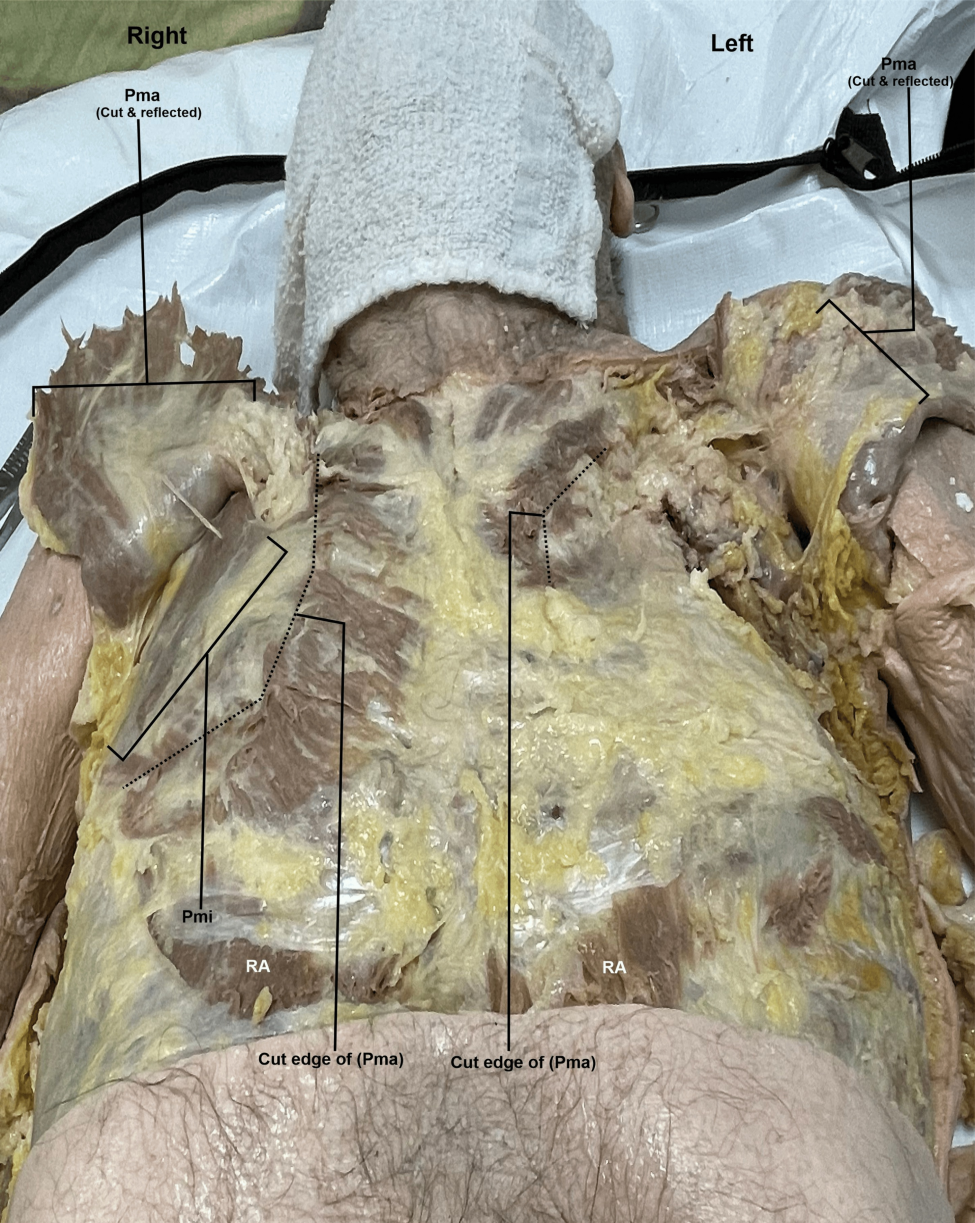
Comparison of the deep dissection of the right and left pectoral regions and anterior wall of the axilla. The right and left pectoralis major muscles have been cut and reflected. On the right side, a normal size and shape of the pectoralis minor is observed, whereas on the left side, the pectoralis minor is completely absent. Pma: pectoralis major; PMi: pectoralis minor; RA: rectus abdominalis

**Figure 4:**
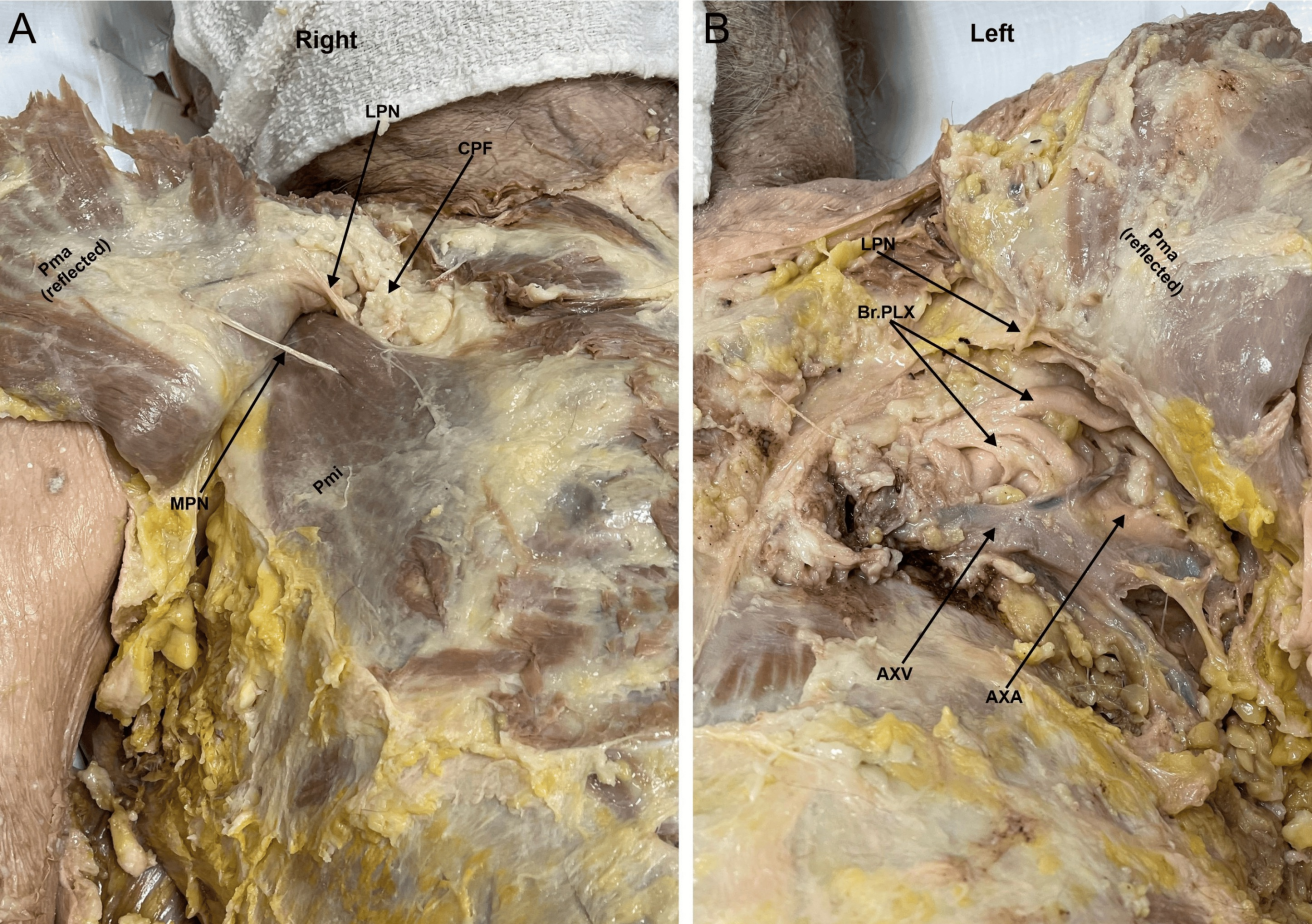
A zoomed-in view of the deep dissection of the right and left pectoral regions and the anterior wall of the axilla. 4A) The right pectoralis minor muscle exhibits a normal size and shape. The muscle is pierced by the medial pectoral nerve. The lateral pectoral nerve is also visible piercing the clavipectoral fascia on the upper border of pectoralis minor to reach the deep surface of pectoralis major. 4B) The left pectoral region shows an absent pectoralis minor and no medial pectoral nerve. The axillary contents (axillary vein and brachial plexus) are visible right under the pectoralis major. The lateral pectoral nerve is also visible diving into the deep surface of the pectoralis major muscle. AXV: axillary vein; AXA: axillary artery; Br. PLX: brachial plexus; CPF: clavipectoral fascia; LPN: lateral pectoral nerve; MPN: medial pectoral nerve; Pma: pectoralis major; PMi: pectoralis minor

During a routine dissection of the musculoskeletal system of a 98-year-old male cadaver, we noted that the PM muscles were asymmetrical on the right and left sides of the body. With careful examination, we realized that the sternocostal head of the left PM muscle was extremely rudimentary compared to a normal and extensive sternocostal head of the right PM (Figures 1-2). The right PM was fully developed and had both the clavicular head and sternocostal head at normal size, covering the entire pectoral region and the anterior wall of the axilla (Figures 1-2). When the PM muscles were cut and reflected on both sides, we noted that the PMi muscle was completely absent on the left side, and the space normally occupied by the muscle was filled with fat covering the contents of the axilla. In contrast, the right PMi had a normal shape and size (Figures 2-3). The right side also has the proper innervation, including the medial and lateral pectoral nerves (Figure 4). The lateral pectoral nerve is still present on the left side, but the medial pectoral nerve is absent (Figure 4). The donor patient does not have any surgical or prior medical history that explains these anatomical variations.

Previous literature has described how PM, PMi, and related nerve anomalies may have functional and clinical consequences. The asymmetry of the pectoral muscles can affect shoulder movements, posture, and overall upper limb function. As a result, activities requiring horizontal adduction, such as throwing and climbing, may be impacted [4]. In a study by Kim et al., it was observed that patients without well-developed or functioning PMi muscles often struggle with overhead movements and exhibit impaired shoulder mechanics [9]. The absence of the PMi and the medial pectoral nerve could also hinder scapular stabilization, further affecting arm and shoulder movements [10]. However, as previously mentioned, surrounding muscles may compensate for these deficiencies, resulting in little overall change in function [8]. Despite this, there is limited literature exploring the functional implications of the absence of pectoralis muscles [8].

Cosmetic changes such as a flattened chest wall or asymmetry may also be present. These aforementioned functional implications could impact surgical planning for reconstructive procedures involving the chest wall or axilla (breast augmentation, flap surgeries, and treatment of axillary surgeries) [4]. Awareness of such variations is important in surgery to avoid complications during chest wall reconstruction, optimize implant positioning, and anticipate potential neurovascular entrapments or functional deficits [4].

## Discussion

The PM and PMi muscles begin development during the fourth to eighth week of gestation. These muscles originate from the mesoderm, specifically the paraxial mesoderm, which surrounds the neural tube. The mesoderm is one of the three germ layers in the developing embryo, and it develops into skeletal muscles. The paraxial mesoderm forms somites on either side of the neural tube. Somites are specialized blocks of cells that then differentiate into the sclerotome, myotome, and dermomyotome. The dermomyotome further divides into the ventral hypomere and the dorsal epaxial division, contributing to general muscle development. The hypomere develops into the prepectoral mass - then cleaves into the superficial entopectoral sheet (developing into PM) and deep entopectoral sheet (developing into PMi) [4]. The muscle mass is attached to the clavicle and distally attaches to the sternum and ribs [1]. Anomalies typically involve a decrease in size or absence of the sternal portion of the PM muscle, whereas the clavicular portion of the PM muscle is usually present and less frequently affected [11].

Variations in the origin and insertion of the PM are seen in up to 23% of the population. Potential variation in origin can include attachment at a more distal point in the shoulder. For example, the PMi has been observed to originate from the glenohumeral joint capsule superior to the coracoid process of the scapula [4]. Anomalies in insertion have manifested in various costal attachments, ranging from the first intercostal aponeurosis to the sixth rib [4]. In our case, the left PM is underdeveloped and smaller than the PM on the right side (Figure 1). The left PM also lacks a medial pectoral nerve. The left PMi lacks vascularization and nerve supply (Figures 3-4).

A related condition, Poland syndrome, is a rare congenital disorder characterized by the partial or complete absence of one or both PM muscles. It may also involve the absence of the overlying mammary gland and can be associated with anomalies in the chest wall or upper limbs [12]. This syndrome is thought to result from genetic factors and exposure to teratogens during embryogenesis, leading to vascular abnormalities [13]. These abnormalities lead to hypoperfusion [13]. In our patient’s case, we observe aplasia of the PM, hypoplasia of the PMi, unilateral involvement, and the absence of the medial pectoral nerve on the left side, all without relevant medical or surgical history. These findings support a diagnosis of Poland syndrome. However, it is crucial to consider other possible diagnoses, as the etiology of Poland syndrome is multifactorial, unlike conditions with a defined genetic cause [14]. This differentiation is significant when counseling families about potential genetic and related anomalies [14].

There is limited literature on the diagnosis and differential diagnoses of Poland syndrome. Baldelli et al. conducted a review of 33 articles on PubMed and identified only four relevant articles [12]. Our findings align with Poland syndrome, categorized as unilateral PM hypoplasia without additional anomalies [12]. The study discusses that, within this category, it is also important to explore differential diagnoses such as localized lipoatrophy, thoracic scleroderma (which can also present with skin anomalies), trauma or surgical history, and isolated mammary or thoracic asymmetry (which does not involve muscle aplasia or nerve absence) [12]. Another important differential diagnosis is Sprengel deformity, which includes hypoplasia of the serratus anterior muscle along with the congenital elevation of the scapula [12]. According to a study by Baas et al. (2018), pectoral muscle hypoplasia alone should not serve as a definitive diagnostic criterion for Poland syndrome [14]. Baas et al. conducted a systematic literature search of 136 articles describing 672 patients [14]. They found that pectoral muscle hypoplasia and other anomalies with a genetic cause are diagnostic of Poland syndrome [14]. Our patient does not have associated anomalies of syndactyly, brachydactyly, or rib defects, which makes the presentation non-syndromic. Our case may fall under the category of non-syndromic congenital pectoral hypoplasia, as our patient lacks the additional malformations typically associated with Poland syndrome, including chest wall depression, missing or underdeveloped nipples, reduced or absent breast tissue (mammary gland), absence of axillary hair, or dextrocardia [14].

Here, we discuss some previously reported PM and PMi anomalies and compare them with our case. A report by Mosconi and Kamath describes a muscle anomaly observed in a 72-year-old female donor [11]. The PM was absent on the right and poorly developed on the left. PMi and lateral pectoral nerves were well-developed bilaterally, while lateral pectoral nerves were absent on both sides. Haladaj et al. conducted a study examining 40 cadavers of both sexes and found the most common anomaly to be a separate clavicular portion of PM [15]. Unlike some previously reported cases of Poland syndrome, in our case, the PM has both clavicular and sternocostal heads present.

Radiological imaging and a thorough patient history could help confirm the diagnosis [12]. The presence of additional anomalies in the patient may indicate a more complex presentation or an overlapping syndrome [12]. Clinically, signs may include asymmetry in the chest wall, such as flattening or underdevelopment of the axillary fold, and other associated abnormalities in anatomy [12]. Ultrasound is often the first imaging method used to assess the presence and thickness of the muscle tissue [12]. For more detailed visualization, magnetic resonance imaging (MRI) can confirm hypoplasia or the absence of specific muscle portions [12]. Additionally, a CT scan provides a cross-sectional view of the chest wall, which can be particularly useful when planning reconstructive surgery [12].

The functional changes in patients with the absence of the pectoralis muscle are typically minimal [8]. Porcellini et al. explored the impact of the absence of the pectoralis muscles on shoulder kinematics [8]. The findings revealed that the lack of pectoral muscles did not lead to significant changes in the range of motion (ROM) during flexion, extension, abduction, and adduction. However, there was a notable presence of shoulder instability in patients, which was attributed to changes in the actions of the posterior internal rotators, unopposed by the PM muscle, potentially resulting in posterior translation of the humeral head. The rotator cuff remained asymptomatic in these patients, indicating that the absence of the PM and minor muscles does not result in functional deficits for the cuff. Additionally, the study found no significant differences in internal rotation strength between the affected and unaffected sides. One study found when PMi is inserted into the joint capsule, it may cause shoulder pain or a decreased ROM at the shoulder joint [4].

Sanchez et al. noted the variability of the relationship between the coastal origins of PM and minor [16]. Their study described how this variability is an important consideration for cosmetic and reconstructive breast surgery and how a strong understanding of precise relationships is helpful in surgical planning and outcomes [16]. The tendon of the PMi is sometimes used in repairs, including acromioclavicular joint reconstruction and subscapularis tears [4]. In some studies, PMi tendon abnormalities were studied in relation to the absence of a coracohumeral ligament, which serves to stabilize the shoulder, prevent inferior translation, and secure the biceps brachii tendon [4]. A patient lacking a PMi tendon, therefore, may be at higher risk for shoulder injury. In the case of our patient, repair mechanisms based on the PMi would not be available.

Patients with chest wall deformities face challenges related to body image and quality of life, often feeling self-conscious or socially uncomfortable [12]. Treatment options range from non-surgical (conservative) methods to surgical interventions, depending on the deformity’s severity, symptoms, and patient preferences [12]. Surgery may be recommended primarily for aesthetic improvements or alleviating functional issues such as restricted movement or breathing difficulties [12]. According to an article by Romanini et al., reconstruction techniques are guided by the Total Base Number (TBN) classification system and can include chest wall reconstruction by muscle transposition via traditional open surgeries or minimally invasive approaches, which aim to reduce recovery time and scarring [17]. Conservative treatments like bracing, physical therapy, or cosmetic prostheses may be appropriate for less severe cases [12]. Ultimately, the decision to pursue surgery or conservative treatment is based on the severity of symptoms and objective assessments of the deformity [12].

Limitations of our case include limited sample size and an inability to discuss the mobility effects of the variation with the patient. Because the variation is only present in a single donor patient, we lack the ability to compare our anomaly with other anomalies. Since the donor patient only had the variation on one side, we can compare between the right and left sides of the patient to gather additional information. However, since the donor patient is a cadaver used in an educational setting, we do not have access to past medical records or to patient testimony.

## Conclusions

This case report further establishes many types and presentations of PM and minor structural anomalies. Documenting and disseminating these variations in PM and PMi morphology are important in clinical education since they can impact normal function in living patients. These differences in anatomy can manifest as physical limitations in ROM or strength. Insertion and origin differences can also lead to shoulder pain or nerve damage. We recommend further investigation of the effects of PM and minor anomalies on various patient populations to provide more complete future care. Potential future studies could work on establishing the impact of the ROM in the shoulder in a living patient population.
